# The prevalence of severe acute respiratory coronavirus virus 2 (SARS-CoV-2) IgG antibodies in intensive care unit (ICU) healthcare personnel (HCP) and its implications—a single-center, prospective, pilot study

**DOI:** 10.1017/ice.2020.298

**Published:** 2020-06-12

**Authors:** Mohsin Sheraz Mughal, Ikwinder Preet Kaur, Chandler D. Patton, Nagy H. Mikhail, Chairut Vareechon, Kenneth M. Granet

**Affiliations:** Monmouth Medical Center, an affiliate of the RWJ/Barnabas Health System, Long Branch, New Jersey

*To the Editor—*Healthcare personnel (HCP), including practitioners, nursing staff, respiratory therapists, and the prone-positioning team caring for coronavirus disease 2019 (COVID-19) patients in the intensive care unit (ICU) are considered to have a high risk of exposure to severe acute respiratory syndrome coronavirus 2 (SARS-CoV-2). Most patients admitted to the ICU are severely sick and need to be intubated. High-risk procedures for droplet dispersion, including tracheal intubation and tracheostomy tube placement, can be performed in the ICU.^1^ In a community seroprevalence study in Los Angeles County, the prevalence of antibodies to SARS-CoV-2 was 4.65%.^2^ To our knowledge, no other study has addressed the prevalence of subclinical seroconversion of SARS-CoV-2 among HCP in the ICU setting. In this study, we investigated the seroconversion of asymptomatic SARS-CoV-2 infection in ICU HCP exposed to critically ill COVID-19 patients.

## Methods

This single-center, prospective, pilot study was performed in an ICU at a teaching hospital, Monmouth Medical Center in Long Branch, New Jersey. It was approved by our institutional review board. All HCP caring for COVID-19 patients in the ICU setting from March 1, 2020, to April 30, 2020, were eligible for inclusion in the study. A cross-sectional survey questionnaire was utilized to collect demographic characteristics and to exclude HCP who (1) tested positive for SARS-CoV-2 by reverse transcriptase-polymerase chain reaction assay (RT-PCR), (2) had symptoms consistent with COVID-19, or (3) had COVID-19 exposure in a household setting. In total, 134 ICU HCP responded to the survey, and 121 HCP were eligible for SARS-CoV-2–specific IgG antibody testing. Means and interquartile ranges (IQRs) were used for continuous variables. All participants provided written consent. Antibody testing was performed on the sera using a rapid immunochromatography test (STANDARD Q COVID-19 IgM/IgG Duo, SD Biosensor, Suwon-si, Korea) by lateral flow in a Clinical Laboratory Improvement Amendments certified (CLIA), high-complexity laboratory. The manufacturer’s stated sensitivity and specificity for IgG, 15–21 days after symptoms onset are 96.2% and 96.6%, respectively. Blood specimens were drawn from 2 weeks after the specified period commencing May 14, 2020, and ending May 19, 2020.

## Results

Overall, 134 ICU HCP responded to the survey: 75% were women, 47.73% were registered nurses, 9.85% were attending physicians, 26.52% were resident physicians, 6.82% were patient care assistants, 6.82% were respiratory therapists, 1.52% were technicians, and 0.76% were anesthetists. The mean age of the respondents was 39.2 years (IQR, 28–48.5). The mean duration of work was 29.3 days (IQR, 16.0–40.0). Of 134 ICU HCP eligible staff, 13 were excluded and 121 underwent SARS-CoV-2–specific IgG antibody testing. One individual tested positive and 1 test result was inconclusive due to a faulty test kit and was removed from the analysis. In this study, the prevalence of asymptomatic seroconversion was 0.83%.

## Discussion

Information about the prevalence of asymptomatic seroconversion of SARS-CoV-2 in HCP is limited. In a preliminary report released by the Centers for Disease Control and Prevention (CDC), nearly 9,282 HCP have contracted COVID-19, and 27 have died.^3^ Okba et al^4^ demonstrated that most PCR-confirmed SARS-CoV-2 patients seroconverted after 2 weeks of disease onset.^4^ Our study revealed a prevalence of 0.83%, which indicates that seroconversion in ICU HCP was a rare event. These data indicate that proper education and utilization of personal protective equipment (PPE) is effective.^5^ Additionally, ventilated patients have less aerosolization and were housed in a negative-pressure environment in the ICU isolation rooms, which also may have been factors in avoiding transmission to HCP.

Our study has several limitations. It was conducted in a single-center ICU and did not include long-term clinical or laboratory follow-up. Studies with larger sample sizes in different healthcare settings would be useful to validate the clinical impact of our findings.
